# Mini-ACE: Validation Study Among Older People in Long-Term Care

**DOI:** 10.5334/joc.330

**Published:** 2024-01-09

**Authors:** Alexandra Grasina, Helena Espirito-Santo, Laura Lemos, Maria Manuela Vilar, Luís Simões-Cunha, Fernanda Daniel

**Affiliations:** 1 Miguel Torga Institute of Higher Education (ISMT), Coimbra, PT; 2 Centro de Estudos e Investigação em Saúde, Universidade de Coimbra, Coimbra, Portugal; 3 Centro de Investigação em Neuropsicologia e Intervenção Cognitiva e Comportamental, Portugal; 4 Faculdade de Psicologia e de Ciências de Educação da Universidade de Coimbra (FPCEUC), Coimbra, Portugal; 5 Miguel Torga Higher Education Institute (ISMT), Coimbra, Portugal; 6 Research and Development Center of the Military University Institute, Lisboa, Portugal; 7 Centro de Inovação em Biomedicina e Biotecnologia da Universidade de Coimbra, Coimbra, Portugal

**Keywords:** cognitive impairment, long-term care, cognitive assessment, older adults, M-ACE

## Abstract

**Background:**

The Mini-Addenbrooke’s Cognitive Examination (M-ACE) is a valid and reliable tool that accurately differentiates various types of cognitive impairment from Normal-cognition assessed in multiple settings. However, its validity among older individuals in long-term care (LTC) was not yet established. Therefore, we sought to assess the Portuguese M-ACE’s validity, reliability, and accuracy in detecting cognitive impairment no-dementia (CIND) in LTC users.

**Methods:**

A comprehensive assessment was performed on 196 LTC Portuguese users aged ≥ 60 years, among whom 71 had Normal-cognition, and 125 had CIND.

**Results:**

The M-ACE was found to be reliable (McDonald’s ω = .86, Cronbach’s α = .85) and consistent over time (*r* = .72; ICC = .83) and between raters (k = .92). Strong correlations with related measures supported construct validity (both *r* = .67). The M-ACE accurately distinguished CIND from Normal-cognition with a cut-off of 17 points (AUC = 0.81, Sensitivity = 81.7%, Specificity = 74.4%).

**Conclusion:**

Our findings suggest that the Portuguese M-ACE is a valid and reliable cognitive assessment tool for LTC users, allowing for accurate differentiation between CIND and Normal-cognition. Thus, the M-ACE’s use could contribute to the early detection and intervention of cognitive disorders, especially among older adults in LTC.

The Portuguese version of the Mini-Addenbrooke’s Cognitive Examination (M-ACE) has been found to be a reliable and valid tool for assessing cognitive impairment no-dementia (CIND) among older adults in long-term care (LTC) settings. The M-ACE accurately distinguishes CIND from normal cognition, which could facilitate early detection and intervention of cognitive disorders in LTC, benefiting both the individuals and the LTC community.

## Introduction

Cognitive impairment and dementia are significant public health concerns, especially among the institutionalized older population (Andrade et al., 2017; Camacho-Conde & Galán-López, 2020; Daniel et al., 2019; Kao et al., 2022; M. Petersen, 2019). Given the subtlety of cognitive decline, neuropsychological tests are critical for clinical diagnosis and research on aging-related cognitive impairment and disorders (R. C. Petersen et al., 2001; Tangalos & Petersen, 2018). However, the validity of existing neuropsychological tests for older individuals in long-term care (LTC) settings has been scarcely investigated (Chou et al., 2001; Rashedi et al., 2019), resulting in limitations in their use. Moreover, common factors in LTC settings such as low education, advanced age, and depressive and anxiety symptoms (Andrade et al., 2017; Daniel et al., 2015; Espirito-Santo, Pena, et al., 2016; Espirito-Santo & Daniel, 2018; Figueiredo-Duarte et al., 2021; Kao et al., 2022; Parmelee et al., 1989; Villeneuve et al., 2022) may affect test scores, leading to inaccurate diagnostic conclusions (Marcopulos et al., 1999; Morgan et al., 2019).

To address this issue, it is necessary to investigate the validity of existing neuropsychological tests in LTC settings. While many neurocognitive tests are available (Aslam et al., 2018; Wang & Dong, 2018), only a few tests, such as the Mini-Mental State Examination (Folstein et al., 1975), the Short Test of Mental Status (Kokmen et al., 1991), and the Montreal Cognitive Assessment (Nasreddine et al., 2005) serve as shortened batteries that cover most key cognitive domains (Lonie et al., 2009). Selecting neuropsychological tests requires a balance between comprehensive assessment and practical constraints, and these shortened batteries may reduce the potential for misdiagnosis without increasing the time burden (Marcopulos et al., 1999; Morgan et al., 2019). This consideration is critical for older participants who may experience high levels of fatigue and lack of energy (e.g., Zengarini et al., 2016). These challenges are amplified in LTC settings, where constraints such as limited staffing, high patient-to-caregiver ratios, and often unpredictable care needs further impose significant time constraints and resource limitations (Daniel et al., 2015; Figueiredo-Duarte et al., 2021).

The Mini-Addenbrooke Cognitive Examination (M-ACE) is a shortened battery that could be a helpful tool due to its short administration time, low cognitive demand, and high sensitivity in detecting cognitive dysfunction (Cao et al., 2022; Kaczmarek et al., 2022). Compared to the full Addenbrooke’s Cognitive Examination, the M-ACE is more practical for settings with time constraints as it requires less administration time while still covering essential cognitive domains. The M-ACE covers four cognitive domains [attention/orientation, memory, verbal fluency, and visuospatial function] with five items and is scored out of 30. The M-ACE was more sensitive than the MMSE and less likely to have ceiling effects, making it a valuable tool in assessing mild cognitive impairment and dementia (Hsieh et al., 2015).

The M-ACE has been translated and validated in several languages, with validation studies conducted in various countries, such as Thailand (Charernboon, 2019), Poland (Kaczmarek et al., 2022), Greece (Kourtesis et al., 2020), the United Kingdom (Larner, 2019), Hungary (Lucza et al., 2018), Brazil (Miranda et al., 2018), China (Pan et al., 2022), Egypt (Qassem et al., 2021), and Japan (Senda et al., 2020). The Portuguese version of the M-ACE, which was extracted from the ACE-III, has also been validated and shown good psychometric properties (Peixoto et al., 2019).

Although the M-ACE is widely used in various clinical and non-clinical settings to assess cognitive function (Hobson et al., 2016; Kaczmarek et al., 2022; Larner, 2019; Ridley et al., 2018; Senda et al., 2020; Trunfio et al., 2022), no research has been conducted on its validity among individuals in LTC settings.

Through our 13-year research experience in Portuguese LTC settings, we have observed that diagnosing cognitive impairment in these facilities can be challenging and incorrect due to limited resources, and the stricter diagnostic criteria for conditions such as mild cognitive impairment (MCI) and dementia may not be suitable. This diagnostic challenge is particularly problematic as sometimes no physician exists or only one general practitioner is available with few resources to perform a comprehensive evaluation. This situation is explained by the fact that most of these facilities are privately-owned social solidarity institutions, financially supported by the Portuguese government (Daniel et al., 2016) that only require one nurse per every 40 residents, and a general practitioner is not considered part of the required staff.

Considering the resource constraints in LTC facilities, *cognitive impairment with no dementia* (CIND) may provide a more suitable framework for diagnosing individuals with cognitive impairments. CIND is a condition of lower cognitive performance than expected for age and education level without meeting the criteria for dementia (Graham et al., 1997; Tuokko et al., 2001). Subjects with CIND exhibit neuropsychological scores that overlap with both normal and demented subjects, presenting a wide range of cognitive symptoms such as memory, attention, language, visuospatial abilities, and executive function problems (Tuokko et al., 2001). This inclusive diagnosis encompasses individuals with cognitive symptoms of diverse etiologies, such as cerebrovascular disease, depression, systemic vascular disease, and other psychiatric conditions (Graham et al., 1997; Tuokko et al., 2001), which are common in LTC settings (Camacho-Conde & Galán-López, 2020; Daniel et al., 2015; Figueiredo-Duarte et al., 2021; Kilian et al., 2018). The broader conceptualization of CIND has been shown to help capture individuals with cognitive impairments due to heterogeneous etiology and to identify a larger representation of individuals at higher risk for developing dementia in the future (Ritchie & Tuokko, 2011). Thus, studying the LTC population with CIND may elucidate the clinical correlates of progression to dementia among those at-risk. Therefore, the M-ACE could be a valuable tool in detecting CIND in this at-risk population. Hence, this study aimed to examine the psychometric properties of the Portuguese version of the M-ACE, including its validity (convergent, concurrent, and divergent) and reliability (McDonald’s omega, Cronbach’s alpha, test-retest, inter-rater) and its accuracy in predicting CIND in a sample of Portuguese LTC users. Additionally, we examined the ability of the M-ACE to differentiate between individuals with CIND and those with Normal-cognition (discriminant validity) and tested the known-group hypothesis.

## Methods

### Transparency and Openness

The de-identified data, and analytical code supporting this study are housed in the Miguel Torga Institute of Higher Education Repository. While these are not publicly accessible due to ethical and institutional guidelines, they can be requested via the repository at https://repositorio.ismt.pt/handle/123456789/1504. Both versions of the M-ACE used in this study can be found at a separate link within the same repository: https://repositorio.ismt.pt/handle/123456789/1555.

### Study Design

This cross-sectional study was conducted as part of the “Aging Trajectories” project, which is affiliated with the Miguel Torga Institute of Higher Education (PTDC/PSI-PCL/117379/2010) and was approved by its Ethics Committee (CE-P11-18) as well as by the directors the LTC facilities.

### Participants and Procedures

A total of 586 participants were recruited from LTC facilities in the central region of Portugal between October 2019 and August 2022. Participants had to meet the inclusion criteria of being aged 60 years or older, fluent in Portuguese, and cognitively and physically able to understand the assessment instructions and provide written informed consent. Exclusion criteria included a documented history of severe psychiatric, neurological, or other systemic physical disorders, substance abuse, recent hospitalization, and hearing or visual impairment that would have clearly interfered with the assessment. Exclusions were verified with general practitioners, nurses, and/or center directors. Out of the total, 73 were initially excluded based on these criteria, and an additional 267 were not able to participate due to restrictions related to the COVID-19 pandemic.

After these initial exclusions, participants underwent a comprehensive assessment covering cognitive, emotional, functional, and executive domains, which included four neuropsychological tests, two questionnaires, and an interview in the present study. Post-assessment, 23 more individuals were excluded due to meeting criteria for a neurocognitive disorder, which was defined as having scores below the cut-off on one or two of the multiple-cognitive domain screening tools used, in addition to demonstrating functional impairment, as outlined in the DSM-5™ (American Psychiatric Association, 2013). An additional 17 participants were excluded for inability to perform the clock-drawing M-ACE, and ten others opted out the M-ACE assessment. It is worth noting that all ten of the participants who refused had no formal education.

Of the remaining 196 participants, 68.9% were women, with a mean age of 82.54 years (*SD* = 8.19; range: 60–96), education of 3.94 years (*SD* = 3.19; range: 0–19), and a mean length of institutionalization of 29.62 months (*SD* = 37.27; range: 1.00–219.00). Of the total, 125 presented CIND (63.8%), and 71 had Normal-cognition (36.2%). The CIND diagnosis was based on the identification of individuals with lower scores in at least one tool assessing multiple cognitive domains or self-reported difficulties in cognition, an approach consistent with the definition of CIND (Graham et al., 1997; Tuokko et al., 2001). All of the normal-cognition participants had scores above the cut-off scores in both cognitive assessments and reported no difficulties in cognition.

After three months, 51 participants (26.0%) were reassessed with a slightly different version of the test.

### Measures

#### Sociodemographic Questionnaire

The sociodemographic questionnaire inquired about age, birthdate, age, sex, education (years and level), civil status, nationality, district of residence, institution, and length of institutionalization.

#### Mini-Addenbrooke – Portuguese version (M-ACE)

Following Erkut (2010) guidelines, the M-ACE was translated into Portuguese by an expert translator who took into account the cultural and linguistic specificities of the language. To ensure accuracy, the translated version was subsequently back-translated by a second translator with extensive proficiency in English. The research team compared the translated and back-translated versions and verified no conceptual differences between the versions. Next, to assess the instructions’ clarity and the items’ fluency and comprehensibility, ten older adults completed the M-ACE (Appendix 1) and were asked to provide feedback. No difficulties or inconsistencies were reported. A slightly different version was created for retest purposes, featuring changes in the two memory items domain to minimize the impact of rehearsal effects.

#### Mini-Mental State Examination (MMSE)

The MMSE [Folstein et al. (1975), Guerreiro et al. (1994)] is an 11-item tool that evaluates cognitive functioning, grouped into four cognitive domains: Orientation (temporal and spatial), Memory (immediate and short-term), Attention (calculation), Language (naming, following a 3-step command, verbal repetition, reading, and writing), and Visuospatial Skills (constructive ability). The total score ranges from 0 to 30 points (better cognitive functioning). We used MMSE to test M-ACE convergent validity, confirm dementia diagnosis, and identify participants with CIND using the cut-off scores proposed by Morgado et al. (2009). Cronbach’s alpha was .84, and McDonald’s ω was .83 in the current study.

#### Frontal Assessment Battery (FAB)

The FAB [Dubois et al. (2000); Espirito-Santo, Queiroz-Garcia et al. (2016)] is a six-item tool that assesses executive functioning through tasks that refer to different frontal lobe functions, which can be grouped into three subscales: FAB-Linguistic (similarities and phonemic verbal fluency), FAB-Planning (Luria motor sequences and conflicting instructions), and FAB-Inhibitory (go-no-go and prehension behavior). The maximum score is 18, with higher scores indicating better performance. We used FAB to test M-ACE concurrent validity and to confirm the absence of dementia. A cut-off score of 7 (Espirito-Santo, Queiroz-Garcia, et al., 2016) was used to classify participants with CIND. The FAB demonstrated good internal consistency (Cronbach’s α = .72, McDonald’s ω = .85).

#### The 12-item World Health Organization Disability Assessment Schedule 2.0 (WHODAS 2.0)

To further support dementia and CIND diagnoses, we used the interview version of WHODAS 2.0, which measures disability over the preceding 30 days and is based on the International Classification of Functioning, Disability, and Health (ICF) framework (World Health Organization, 2023). The WHODAS 2.0 consists of six subscales, each containing two questions: Cognition (WHODAS-Cog), Mobility (WHODAS-Mob), Self-care (WHODAS-Car), Getting Along with People (WHODAS-Peop), Life Activities (WHODAS-Act), Participation (WHODAS-Par). This five-minute tool uses an ordinal scale ranging from 0 (*none*) to 4 (*extreme/cannot do*) for each response choice. The subscale scores are calculated by summing the two items within each subscale, and the total score is a percentage (∑item scores / 48×100), with scores ranging from 0 (no disability) to 100 (full disability) (Üstün et al., 2010). The severity of total disability scores is categorized based on the ICF ranges, with a score of 25 or greater indicating disability based on the WHODAS ICF (World Health Organization, 2023). Reliability was good in the current study (Cronbach›s α = .93, McDonald›s ω = .94). The WHODAS-Cog was also employed to categorize participants with CIND.

#### The 8-item Geriatric Depression Scale (GDS-8)

The GDS-8 (Figueiredo-Duarte et al., 2021; Yesavage et al., 1983) was used to assess depressive symptoms in our sample. The GDS-8 consists of eight items that are answered with “yes” or “no” and refer to the last week. Scores range from zero to eight, with higher scores indicating greater severity of depressive symptoms. The internal consistency of the GDS-8 was high in our study (Cronbach’s α = .88). Given that depression is a different construct of cognitive functioning, although moderate correlations are expected (e.g., Figueiredo-Duarte et al., 2021; Sumiyoshi et al., 2019), the GDS-8 was used to assess divergent validity.

### Statistical analysis

To ensure sufficient statistical power for the planned analyses, we conducted preliminary power analyses using G*Power software (https://bit.ly/3FZArXO). Based on an alpha of .05 and a power of .80, we determined that a sample size of > 50 per group, or > 104 in total, would be needed for Student’s *t*-tests, correlation, and regression analyses, assuming effect sizes of *d* = 0.5, *r* = .5, and *f*^2^ = 0.2, respectively. For Receiver Operating Characteristic (ROC) analysis, we aimed to include at least 20 positive and four negative cases to achieve a power of .80 (computation with http://www.biosoft.hacettepe.edu.tr/easyROC/) based on the reported prevalence rates of cognitive impairment in Portugal (41.7% and 16.9%) in previous studies (Daniel et al., 2019; Pais et al., 2020) and for an area under the curve (AUC) between .86–.89 (Kaczmarek et al., 2022; Senda et al., 2020). Thus, our final sample size of 196 participants exceeded the determined requirements for statistical power, ensuring adequate power for our planned analyses.

The analytical strategy included, firstly, comparing demographic variables between CIND–Normal-cognition groups using Student’s *t*-tests and chi-square and examining descriptive statistics and frequency distributions. Additionally, to compare the mean scores of the M-ACE domains, we used the percent of the maximum possible (POMP) approach (Cohen et al., 2010), which involves calculating the proportion of the total possible score that was achieved by each participant [(mean score – minimum possible score)/(maximum possible score – minimum possible score) x 100]. We also used the chi-square test (with phi coefficients computed for effect size) to analyze the association between failures-correct answers for each M-ACE task and groups (CIND vs. normal-cognition).

Second, the internal consistency reliability was evaluated using Cronbach’s alpha coefficient and McDonald’s omega (.70–.90, Streiner, 2003), along with corrected item-total correlations (*r*_b_ > .40, Ware & Gandek, 1998). Test-retest reliability was assessed with Pearson and intraclass correlation coefficients (ICC > .75, Weir, 2005). Given that only studies with other ACE versions computed test-retests (Fang et al., 2014; Pan et al., 2022; Suriyakumara et al., 2019), their values were used as a range for comparison (.93–.99). Cohen’s kappa was computed to assess inter-rater reliability between two independent scorers, with values between .93–.90 used as a reference based on previous works with other ACE versions (Fang et al., 2014; Matias-Guiu et al., 2015; Pan et al., 2022; Suriyakumara et al., 2019). However, due to the nature of the Clock-drawing and the potential for some subjectivity in its scoring, we performed Cohen’s kappa coefficient calculation only for this task (the scoring of the other tasks in the M-ACE is straightforward and does not require a unique scoring approach). To assess construct validity, we employed Pearson’s correlation analysis with 95% confidence intervals (CI) obtained from 1000 bootstraps to examine the associations between M-ACE and established cognitive tools, including MMSE (convergent validity), FAB (concurrent validity), and GDS-8 (divergent validity). Based on prior findings with MMSE (Charernboon, 2019; Hobson et al., 2016; Hsieh et al., 2015; Kaczmarek et al., 2022; Kourtesis et al., 2020; Miranda et al., 2018; Qassem et al., 2021), correlation coefficients between .64–.94 were considered optimal. For divergent validity, we expected lower correlation coefficients, indicating that the M-ACE measures a distinct construct from depressive symptoms.

Third, the diagnostic accuracy of the M-ACE classification was evaluated by calculating the area under the receiver operating characteristic curve (AUC of the ROC), along with the sensitivity and specificity, using the cut-off scores for MMSE and /or for FAB to classify participants with CIND. Considering previous studies with subjects with MCI (Charernboon, 2019; Hobson et al., 2016; Kaczmarek et al., 2022; Larner, 2020; Lucza et al., 2018; Pan et al., 2022; Senda et al., 2020; Trunfio et al., 2022; Yang et al., 2019), an AUC between .77–.89 was considered a quality marker for the M-ACE classification performance. The M-ACE cut-off score with the highest Youden index (> .8 high, .4–.6 moderate level of separation) was used as the optimal cut-off (Fluss et al., 2005). Fourth, to compare M-ACE scores between CIND and normal-cognition groups, we conducted a Student *t*-test and plotted a bar graph, with the mean scores represented as the bar height and the 95%CI interval around the mean represented as the error bars.

Finally, the influence of sociodemographic variables on individual M-ACE scores was investigated using multivariable regression analysis.

JAMOVI 2.3.21 was used for the analyses, which is a free and open-source statistical software that offers a user-friendly interface for conducting data analyses (JAMOVI Development Team, 2021).

## Results

### Preliminary analysis

There were no statistically significant differences in age or education years between CIND and Normal-cognition groups (*t*_(194)_ = 1.62, *p* > .05, *d* = 0.24; *t*_(194)_ = 0.34, *p* > .05, *d* = 0.05) and no association with gender (χ^2^_(1)_ = 0.08, *p* > .05).

Table 1 presents the descriptive statistics for the study instruments. The *z*-scores for skewness and kurtosis indicated normal distributions of most scores, except for M-ACE Attention/Orientation, MMSE total score, Attention, Orientation, Language, and Visuospatial.

**Table 1:** Descriptive and Correlation Analyses for Study Variables.


VARIABLES	*M*	*SD*	PEARSON CORRELATIONS				

			1	2	3	4	5

Mini-Addenbrooke (M-ACE)	15.33	6.34	—				

M-ACE Attention/Orientation	2.75	1.32	.68***	—			

M-ACE Memory	7.48	3.46	.89***	.47***	—		

M-ACE Fluency	2.71	1.67	.73***	.42***	.48***	—	

M-ACE Visuospatial	2.39	1.59	.72***	.42***	.48***	.46***	—

Mini-Mental State Examination (MMSE)	22.41	4.99	.67***	.69***	.51***	.48***	.48***

MMSE-Attention	2.92	2.04	.41***	.39***	.31***	.34***	.30***

MMSE-Orientation	7.82	2.23	.65***	.75***	.49***	.44***	.46***

MMSE-Memory	4.34	1.25	.37***	.31***	.32***	.26***	.24***

MMSE-Language	7.12	1.09	.38***	.40***	.30***	.24***	.27***

MMSE-Visuospatial Skill	0.22	0.41	.35***	.30***	.22***	.31***	.37***

Frontal Assessment Battery (FAB)	9.37	3.80	.67***	.55***	.56***	.46***	.52***

FAB-Linguistic	2.68	1.58	.61***	.50***	.46***	.51***	.49***

FAB-Planning	2.81	1.84	.53***	.42***	.48***	.30***	.40***

FAB-Inhibitory Control	3.87	1.32	.44***	.40***	.36***	.28***	.35***

Geriatric Depression Scale-8-Items	4.26	2.77	-.11	-.16*	-.02	-.16*	-.09


*Note*: Correlations between similar domains are highlighted in grey. *** *p* < .001. ^*^ *p* < .05.

The POMP scores for the M-ACE domains range suggested a considerable variation in performance across the domains. Specifically, the POMP scores were as follows: Attention, 68.8%; Memory, 53.4%; Verbal Fluency, 38.7%; and Visuospatial, 47.8%.

**Figure 1:**
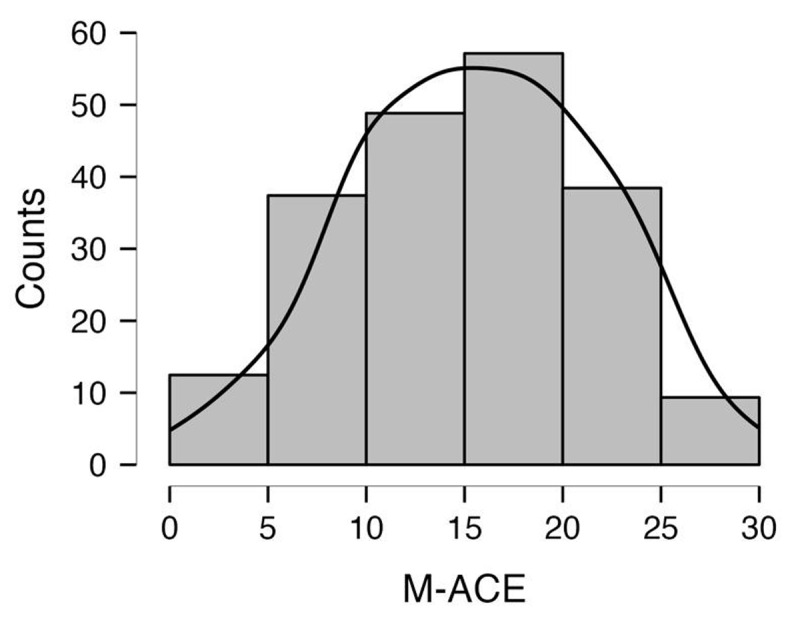
Distribution of M-ACE Scores in Individuals with CIND and Normal Cognition. *Note*: M-ACE = Mini-Addenbrooke Cognitive Examination; CIND = cognitive impairment no-dementia.

The M-ACE scores exhibited little evidence of ceiling and floor effects (Figure 1).

Table 2 displays the proportion of Mini-Addenbrooke’s tasks failures in the CIND and Normal-cognition groups. The CIND group had a significantly higher proportion of failures than the Normal-cognition group in almost all tasks. The greatest differences between the two groups were observed in the Memory Recall and Attention/Orientation domains. Specifically, the highest proportions of failures in the CIND group were observed in the Month Day (Attention/Orientation domain), Address Street, and Address Town tasks (Memory Recall domain) as indicated by their phi coefficients. The performance contrast is further highlighted by the substantial disparities in the proportions of task failures between the two groups.

**Table 2:** Proportions of Mini-Addenbrooke’s Tasks Failures in CIND and Normal-cognition Groups.


TASKS	CIND (*n* = 125)		NORMAL-COGNITION (*n* = 71)		χ^2^	ϕ

	*n*	%	*n*	%		

Attention/Orientation						

Weekday	35	28.0	4	5.6	14.21***	.27

Month day	75	60.0	13	18.3	31.81***	.40

Month	24	19.2	4	5.6	6.81***	.19

Year	60	48.0	13	18.3	17.08***	.30

Memory						

First name	27	21.6	3	4.2	10.54***	.23

Last name	29	23.2	6	8.5	6.72***	.19

Address street	36	28.8	13	18.3	2.66	.12

Address name	59	47.2	25	35.2	2.66	.12

Address number	46	36.8	19	26.8	2.06	.10

Address town	47	37.6	23	32.4	0.53	.05

Address district	35	28.0	8	11.3	7.40***	.19

Semantic Fluency						

<5 words	20	16.0	2	2.8	7.90***	.20

Clock Drawing						

Circle	24	19.2	2	2.8	10.56***	.23

Numbers (0 points)	60	48.0	11	15.5	20.71***	.33

Hands (0 points)	97	77.6	34	47.9	18.04***	.30

Memory recall						

First name	82	65.6	24	33.8	18.44***	.31

Second name	90	72.0	30	42.3	16.88***	.29

Address street	99	79.2	26	36.6	35.54***	.43

Address street name	115	92.0	56	78.9	7.01***	.19

Address number	104	83.2	36	50.7	23.43***	.35

Address town	97	77.6	27	38.0	30.51***	.39

Address district	74	59.2	20	28.2	17.47***	.30


*Note*: *N* = 196. CIND = cognitive impairment no-dementia. *** *p* < .001.

### Reliability and Construct Validity

The internal reliability of the M-ACE was found to be high, with point estimates of .86 for McDonald’s ω (95%*CI*: .82–.88) and .85 for Cronbach’s α (95%*CI*: .82–.88).

Test-retest reliability was good (*r* = .72, ICC = .83, 95%*CI*: .77–.87), with no significant differences between the initial and retest scores (*M* = 15.51, *SD* = 7.01; *t*_(50)_ = 0.71, *p* = .483, *d* = 0.10).

The agreement between raters regarding the clock-drawing task was almost perfect (Cohen’s unweighted kappa = .92, 95%*CI*: .87–.97).

The convergent validity was confirmed for the total score and Orientation domain of the M-ACE as evidenced by strong positive correlations between M-ACE and MMSE and between their respective Orientation-Attention domains (CI95%: .67–.82, .60–.75). Concurrent validity was demonstrated between M-ACE and FAB. Refer to Table 1 for complete correlation results. Furthermore, a moderate negative correlation was found between the M-ACE and WHODAS-Cog (*r* = -.37, *p* < .001).

In what concerns divergent validity, the correlation between the M-ACE and the GDS-8 was weak and negative.

### Predictive Validity

**Figure 2:**
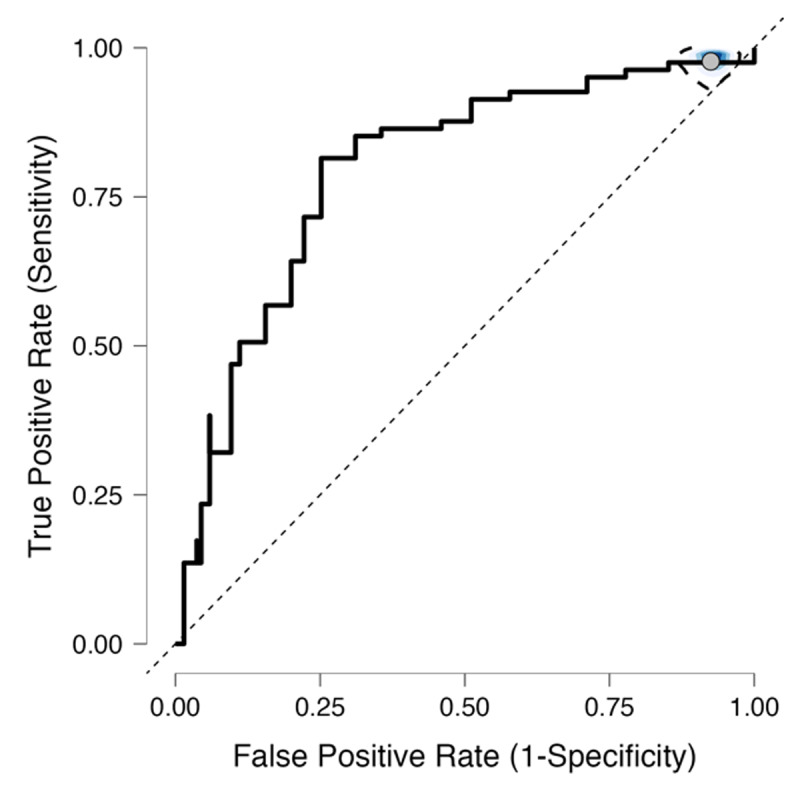
ROC Curve for the M-ACE in Differentiating CIND vs. Normal-cognition. *Note*: M-Addenbrooke Cognitive Examination; CIND = cognitive impairment nodementia.

ROC analysis revealed that the M-ACE demonstrated moderate diagnostic accuracy (Youden’s index = .56) with good discriminatory power (AUC = .81) for differentiating between individuals with CIND and normal cognition. The optimal cut-point of 17 yielded a sensitivity of 81.7%, specificity of 74.4%, positive predictive value of 64.4%, and negative predictive value of 87.7% (Figure 2).

**Figure 3:**
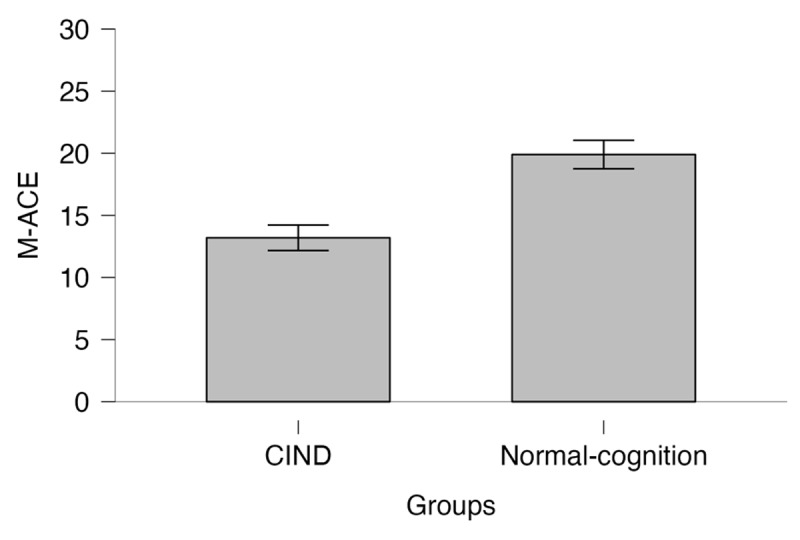
Bar plot of M-ACE Mean Scores in CIND and Normal-cognition Groups with 95%CI. *Note*: M-Addenbrooke Cognitive Examination; CIND = cognitive impairment no-dementia.

A significant difference was observed in the total M-ACE score between the Normal-cognition group (*M* = 19.90, *SD* = 4.84) and the CIND group (*M* = 13.20, *SD* = 5.79) with a large effect size (*t*_(194)_ = 8.25, *p* < .001, Cohen’s *d* = 1.23). Figure 3 presents the bar plot of mean scores with 95%CI.

### Known group hypothesis

A multivariable regression analysis was performed to test the known group hypothesis. Initially, collinearity diagnostics were conducted to ensure the suitability of the data for regression analyses, revealing no issues among the control variable and the predictors (tolerance > 0.01, VIF < 10). Normality was assessed through histograms and Q-Q plots of residuals, which were deemed acceptable. While some outliers were identified in the scatterplots of the standardized residuals, with Mahalanobis distance values above the critical value for three independent variables, the low Cook’s Distance value (0.28) indicated that only a few points had undue influence (Tabachnick & Fidell, 2014). The results indicated a significant association between M-ACE and demographic variables (*R*^2^ = .08, *R*^2^_adjusted_ = .07, *F*_(3, 192)_ = 5.91, *p* < .001). However, the coefficients showed that only years of education (β = 0.24, *t* = 3.26, *p* < .001) had a significant positive association with M-ACE scores, while sex (β = -0.0007, *t* = 0.10, *p* = .919) and age (β = -0.11, *t* = 1.45, *p* =.149) did not.

## Discussion

The aim of this study was to examine the psychometric properties of the Portuguese version of the M-ACE in detecting cognitive impairment in at-risk LTC users. To our knowledge, this is the first study to evaluate the psychometric properties of the M-ACE in a Portuguese sample of the LTC population. The results of our study suggest that the M-ACE is a reliable and valid tool for assessing cognitive impairment in at-risk LTC users in Portugal.

Our preliminary findings suggest a considerable variation in performance across the M-ACE domains in both groups, with the Attention/Orientation and Verbal Fluency domains exhibiting the highest and lowest mean scores, respectively. These results are not surprising considering the assessment context. In LTC settings, users are often exposed to reminders of the date (Attention subscale questions about temporal orientation) and typically have mainly low levels of education (Daniel et al., 2019; Figueiredo-Duarte et al., 2021; Lemos et al., 2019). Furthermore, the M-ACE scores exhibited little evidence of ceiling and floor effects, indicating sensitivity to differences in cognitive ability among LTC users. Moreover, our findings also suggest that the M-ACE is able to differentiate between individuals with CIND and those with Normal-cognition across all tasks, which is consistent with previous research demonstrating that deficits in Memory Recall, Attention/orientation, Verbal Fluency, and Visuo-constructive function are common in individuals with early cognitive impairment (Apolinario et al., 2016; Grober et al., 2019; Sabahi et al., 2022; Saunders & Summers, 2011; Stephens et al., 2004; Tamaru et al., 2023; Valencia & Lehrner, 2021; Zhou & Jia, 2009). However, the most significant differences between the two groups were observed in the Month Day task of the Attention/Orientation domain and Address Street and Address Town of the Memory Recall domains, suggesting that these tasks may be particularly useful in predicting dementia and warranting further investigation.

Regarding reliability, the internal consistency of the M-ACE was high, with strong estimates for McDonald’s ω and Cronbach’s α. Our findings are in line with previous research in different populations demonstrating the strong internal consistency of the M-ACE and its reliability across different cultures and languages (Charernboon, 2019; Hsieh et al., 2015; Kourtesis et al., 2020; Matias-Guiu et al., 2015; Miranda et al., 2018; Pan et al., 2022; Peixoto et al., 2021). Our study also found good test-retest reliability, indicating adequate consistency over time. Additionally, we found no significant differences between the initial and retest scores, further supporting the stability of the M-ACE as a measure of cognitive function in this population. Moreover, the inter-rater agreement was almost perfect. Although there has been limited research on the test-retest reliability and inter-rater agreement of the M-ACE, our results align with studies of other versions of ACE, which have shown good stability and rater agreement across different populations (Fang et al., 2014; Matias-Guiu et al., 2015; Pan et al., 2022; Suriyakumara et al., 2019).

In what concerns construct validity, our study found evidence supporting the convergent and concurrent validity of the M-ACE. The convergent validity of the M-ACE was confirmed for the total score and Orientation domain, as evidenced by strong positive correlations between the M-ACE and MMSE and between their respective Orientation-Attention domains. These findings are consistent with previous research demonstrating the convergent validity of the M-ACE with multi-cognitive domain measures in different populations (Charernboon, 2019; Hobson et al., 2016; Hsieh et al., 2015; Kourtesis et al., 2020; Miranda et al., 2018; Pan et al., 2022; Peixoto et al., 2019; Qassem et al., 2021; Yang et al., 2019). However, we found low to moderate correlations between the M-ACE Memory Recall, Fluency, and Visuospatial domains and their corresponding MMSE domains. The differences in the tasks used to assess these domains may account for these results. For example, the M-ACE Memory Recall includes an address to be remembered, which may be harder to recall than the three words used in the MMSE. Conversely, the MMSE assesses language ability through a variety of tasks such as writing a sentence, obeying a written command, naming objects, and obeying a three-step oral command, which may be more challenging for older individuals. Additionally, the M-ACE Visuospatial domain includes the clock drawing task, which may be harder than the pentagons’ drawing task used in the MMSE. Furthermore, our results demonstrated concurrent validity between the M-ACE and FAB, which is a measure of executive function. This finding is consistent with previous research indicating that executive function is related to overall cognitive functioning in old age (Espirito-Santo, Queiroz-Garcia, et al., 2016; Pellas & Damberg, 2021; Stephan et al., 2014). The divergent validity was confirmed by the weak correlation between M-ACE and GDS-8, indicating that the M-ACE measures cognitive function independently of depressive symptoms. Previous research has shown that cognitive function and depressive symptoms correlate moderately (Figueiredo-Duarte et al., 2021; Sumiyoshi et al., 2019), which is in line with our findings, suggesting that the M-ACE can effectively measure cognitive function in older adults, even when depressive symptoms are present. It should be noted that while there is some evidence for a relationship between depression and cognitive impairment, this relationship is thought to be largely indirect, mediated by factors such as inflammation and changes in brain structure and function (e.g., Leonard, 2017).

It is worth noting that the WHODAS-2 cognition subscale is based on an interview format, which relies on the subjective reporting of the individual’s perception of their cognitive abilities. Therefore, the moderate correlation between M-ACE and WHODAS-2-Cog suggests that there is some overlap between objective and subjective measures of cognitive function. However, the fact that the correlation is not stronger may indicate the influence of other factors such as differences in interpretation or self-evaluation of cognitive functioning, denial or lack of awareness of cognitive difficulties, anxiety or depression affecting self-reporting, or the stigma attached to admitting cognitive impairment (Chambers et al., 2022).

The M-ACE demonstrated moderate diagnostic accuracy and good discriminatory power in distinguishing between individuals with CIND and Normal-cognition in LTC facilities, with an optimal cutpoint of 17 yielding a sensitivity of 81.7% and a specificity of 74.4%. These findings are consistent with previous studies validating the M-ACE in different populations, such as community-dwelling older adults (Charernboon, 2019; Kaczmarek et al., 2022; Qassem et al., 2021), and older individuals with HIV neurocognitive disorders (Trunfio et al., 2022). However, we observed a lower cut-off score compared to those previous studies, likely due to the older age, greater cognitive impairment, and functional disabilities of LTC residents. It is important to consider that the optimal cut-off score may vary based on the population and context in which the M-ACE is utilized, as reported previously (Senda et al., 2020; Yang et al., 2019).

Lastly, our results revealed a significant and large difference in the total M-ACE score between the Normal-cognition and CIND groups, which aligns with previous research (Charernboon, 2019; Hobson et al., 2016; Pan et al., 2022; Qassem et al., 2021; Senda et al., 2020). Furthermore, the only significant predictor of M-ACE scores was years of education, which is also in line with previous studies indicating that higher levels of education are related to better cognitive performance (e.g., Stern et al., 2019). This suggests that years of education may be an important factor to consider when interpreting M-ACE scores in older adults.

## Conclusion

Overall, the study’s results suggest that the Portuguese version of the M-ACE is a reliable and valid tool for assessing cognitive impairment in at-risk LTC users in Portugal. Our findings demonstrate that the M-ACE has strong internal consistency, test-retest reliability, inter-rater agreement, and good construct validity. The M-ACE also showed moderate diagnostic accuracy in distinguishing between individuals with CIND and Normal-cognition in LTC facilities. Finally, our results indicate that years of education may be an important factor to consider when interpreting M-ACE scores in older adults.

These findings have important implications for the early detection of cognitive impairment in older adults and can aid in the development of effective interventions to mitigate the negative consequences of cognitive decline in this population.

## Additional File

The additional file for this article can be found as follows:

10.5334/joc.330.s1 Appendix 1. MINI – ADDENBROOKE’S COGNITIVE EXAMINATION.
