# Supplementary material for: Mini-ACE: Validation Study Among Older People in Long-Term Care
Source: J Cogn. 2024 Jan 9;7(1):5. doi: 10.5334/joc.330 (PMC10785962; doi:10.5334/joc.330)
Supplement: Appendix 1. — MINI – ADDENBROOKE’S COGNITIVE EXAMINATION. [file joc-7-1-330-s1.pdf]

Appendix 1

MINI – ADDENBROOKE'S COGNITIVE EXAMINATION

Versão PT A (2019)

Dizer: vamos de novo fazer algumas perguntas, não se preocupe por algumas serem parecidas com outras que já fizemos.

**ATENÇÃO**					
Perguntar: **Qual é o**	Dia da semana	Dia do mês	Mês	Ano	**Atenção** (Pontuação 0 – 4)

**MEMÓRIA**				
Dizer: **Vou dizer-lhe o nome de uma pessoa e a sua direção e gostaria que a repetisse depois de mim. Vamos fazê-lo três vezes para que possa aprendê-la. Voltarei a perguntar-lhe o nome e a direção mais tarde.**<br>*(Cotar somente o terceiro ensaio)*				**Memória** (Pontuação 0-7)
	1º ensaio	2º ensaio	3º ensaio	
António Amaral	___ ___	___ ___	___ ___	
Rua Formosa, 24	___ ___<br>___	___ ___<br>___	___ ___<br>___	
Luso	___	___	___	
Mealhada	___	___	___	

**FLUÊNCIA – ANIMAIS**					
Dizer: **Agora diga o nome de todos os animais que consiga. Tem um minuto. Pode começar.**				**Fluência** (Pontuação 0-7)	
↓	↓	↓	↓	≥ 22	7
				17-21	6
				14-16	5
				11-13	4
				9-10	3
				7-8	2
				5-6	1

				< 5	0
				Total	Corrigido

**DESENHO DE RELÓGIO**		
Fornecer folha em branco e dizer: **Desenhe um relógio com números e ponteiros, a marcar as cinco e dez.**<br>*(Cotação: Círculo = 1; Números = 2; Ponteiros = 2 se tudo correto).*		**Visuoespacial**<br>(Pontuação 0-5)
**MEMÓRIA**<br>Recordação diferida		
Dizer: **Recorda-se do nome e da direção que repetimos no princípio três vezes?**<br>**Diga-me do que se lembra.**<br>*(Atribuir 1 ponto por cada acerto)*		**Memória** (Pontuação 0-7)
António Amaral	___ ___	
Rua Formosa, 24	___ ___ ___	
Luso	___	
Mealhada	___	
**PONTUAÇÃO TOTAL**		/30
